# Dynamic Wavelength‐Selective Diffraction and Absorption with Direct‐Patterned Hydrogel Metagrating

**DOI:** 10.1002/advs.202408960

**Published:** 2024-10-17

**Authors:** Chenjie Dai, Xinglong Li, Wen‐xing Yang, Yan Chen, Dingshan Zheng, Nian Cheng, Tao Shui, Huafeng Zhang, Zhongyang Li

**Affiliations:** ^1^ School of Physics and Optoelectronic Engineering Yangtze University Jingzhou 434023 China; ^2^ Electronic Information School Wuhan University Wuhan 430072 China

**Keywords:** direct patterning, humidity responsive, hydrogel metagrating, tunable absorption, wavelength‐selective diffraction

## Abstract

Hydrogel nanophotonic devices exhibit attractive tunable capabilities in structural coloration and optical display. However, current hydrogel‐based tunable strategies are mostly based on a single physical mechanism, and it remains a challenge to merge multiple mechanisms for active devices with integrated functionalities. Here, a hydrogel metagrating combining Fabry‐Pérot (FP) resonance and diffraction effects is proposed for achieving tunable absorption and dynamic wavelength‐selective beam steering. Through exploiting hydrogel shrinkage under electron‐beam exposure, a hydrogel nanocavity composed of Ag/Hydrogel/Ag three‐layer films can be directly printed with arbitrary patterns, enabling the direct‐pattering technique of metagrating. The hydrogel nanocavity performs as an FP‐type absorber, and its absorption peak rapidly shifts with humidity variation due to the hydrogel layer scaling. The response speed is <320 ms, and the absorption peak shift range is >150 nm. It is further demonstrated that the hydrogel metagrating exclusively deflects light at the resonance wavelength, and its operating wavelength can be actively switched by regulating ambient humidity. The proposed tunable hydrogel metagrating can promote new technologies of tunable metasurfaces for optical filtering, gas sensing, and optical imaging.

## Graphical abstract

A hydrogel metagrating combining Fabry‐Pérot resonance and grating effects is proposed for achieving tunable absorption and wavelength‐selective diffraction. By exploiting hydrogel shrinkage under exposure, a hydrogel nanocavity can be directly printed with arbitrary patterns, enabling the direct‐pattering technique of metagrating. The hydrogel metagrating exclusively deflects light at the resonance wavelength, and its operating wavelength is actively switched by regulating humidity.

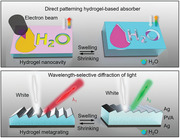

## 1 Introduction

Tunable metasurfaces based on active materials have been steadily researched to endow nanophotonic devices with dynamic optical functionalities,^[^ ^1^, ^2^, ^3^ ^]^ including dynamic structural color,^[^ ^4^, ^5^, ^6^ ^]^ tunable absorption,^[^ ^7^, ^8^, ^9^, ^10^ ^]^ switchable beam steering,^[^ ^11^, ^12^, ^13^, ^14^ ^]^ dynamic holography,^[^ ^15^, ^16^, ^17^, ^18^ ^]^ etc. To explore practical tuning strategies, many efforts have been devoted to designing nanophotonic constructions using various active materials such as transparent conductive materials, liquid crystals,^[^ ^19^, ^20^, ^21^, ^22^ ^]^ phase‐change materials,^[^ ^23^, ^24^, ^25^, ^26^ ^]^ and doped semiconductors.^[^ ^27^ ^]^ Among them, as a representative strategy of nanophotonics for structural color tuning, Fabry‐Pérot (FP) type nanocavities with simplified architectures exhibit dynamic spectral manipulation by utilizing the transformation in the optical properties of active materials,^[^ ^28^, ^29^, ^30^, ^31^, ^32^, ^33^ ^]^ providing a feasible route for commercial applications.

Recently, to improve the continuous tuning ability of FP architecture, humidity‐responsive hydrogel materials with their remarkable swelling properties have been employed to construct hydrogel‐integrated nanocavity.^[^ ^34^, ^35^, ^36^ ^]^ Due to the cavity length scaling from hydrogel expansion, the resonance peak wavelength of the FP nanocavity actively shifts with increasing humidity, thus realizing continuous transmission color exhibitions.^[^ ^37^, ^38^ ^]^ Although the FP resonance‐based hydrogel nanocavities have achieved impressive functionalities in terms of dynamic full‐color displays and optical encryption, it remains a critical challenge to incorporate multiple physical mechanisms into hydrogel nanophotonic device design for multi‐functional integration.

In this work, we experimentally demonstrate a tunable hydrogel‐based metagrating for dynamic wavelength‐selective beam steering by combining the principle of blazed grating effect and FP resonance. We first study the patterning feasibility and dynamic optical properties of a reflective hydrogel nanocavity by using grayscale electron‐beam (e‐beam) lithography. The hydrogel nanocavity is based on the construction of silver‐hydrogel‐silver to form a tunable FP‐type absorber. Owing to the humidity‐responsive swelling of polyvinyl alcohol (PVA) hydrogel, the absorption peak wavelength of hydrogel nanocavity could be actively and rapidly tuned in real‐time by regulating ambient humidity, and the absorption peak shift is >150 nm. Furthermore, the polymer‐shrinkage property of PVA hydrogel under e‐beam exposure provides an effective method to directly print hydrogel nanocavity with arbitrary patterns.^[^ ^35^ ^]^ By employing the direct patterning technique, we further fabricate the hydrogel nanocavity with blazed grating profiles, named hydrogel metagrating. The hydrogel metagrating inherits the wavelength‐sensitive FP resonance due to the cavity effect and offers the ability to deflect light, thereby enabling tunable multi‐functional integration. We envision that the direct‐patterned tunable hydrogel metagrating could facilitate technologies of active nanophotonic devices and suggest potential applications in several fields, including optical sensing, spectral analysis, and optical imaging.

## 2 Results and Discussion

**Figure 1:**
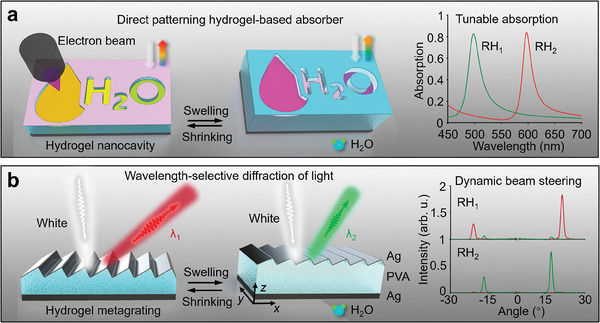
Graphical illustration of direct patterning hydrogel platform for (a) tunable absorption and (b) dynamic wavelength‐selective beam steering. a) The hydrogel nanocavity absorber could be directly fabricated with a designed pattern using grayscale electron‐beam lithography. The ambient humidity variation dynamically tunes the absorption peak of hydrogel nanocavity at visible frequencies due to hydrogel volume swelling. b) The hydrogel blazed metagrating deflects light of a specific wavelength to the far field. The diffraction wavelength of metagrating is actively tuned by the cavity length change from hydrogel inflation.

**Figure** **1** schematically represents the direct‐patterning technique of hydrogel nanocavity and its versatile functionalities for tunable absorption and dynamic wavelength‐selective beam steering. The top and bottom Ag‐layer thicknesses of hydrogel nanocavity are set as 22 and 100 nm to ensure it operates as a typical FP‐type absorber. Since high‐energy radiations could crosslink polymer chain ends without adding crosslinkers^[^ ^39^, ^40^ ^]^ and induce volume shrinkage,^[^ ^41^ ^]^ under e‐beam irradiation, the hydrogel layer shrinks accordingly with the exposure dose variation, enabling the feasibility of arbitrarily patterning hydrogel absorbers. As the ambient relative humidity (RH) increases, the resonance absorption peak of nanocavity is dynamically tuned due to the hydrogel thickness swelling (Figure 1a). By utilizing a gradient‐distributed dose layout to expose the hydrogel nanocavity, the hydrogel metagrating with a blazed grating profile can be fabricated to deflect light. Due to the frequency‐dependent resonance of FP cavity, the hydrogel metagrating deflects the light of resonant wavelength to specific angles. In contrast, light of other wavelengths is vertically reflected under normal incidence conditions (Figure 1b). Moreover, the operating wavelength of hydrogel metagrating could be tuned by the expansion of hydrogel layer, enabling dynamic wavelength‐selective beam steering.

**Figure 2:**
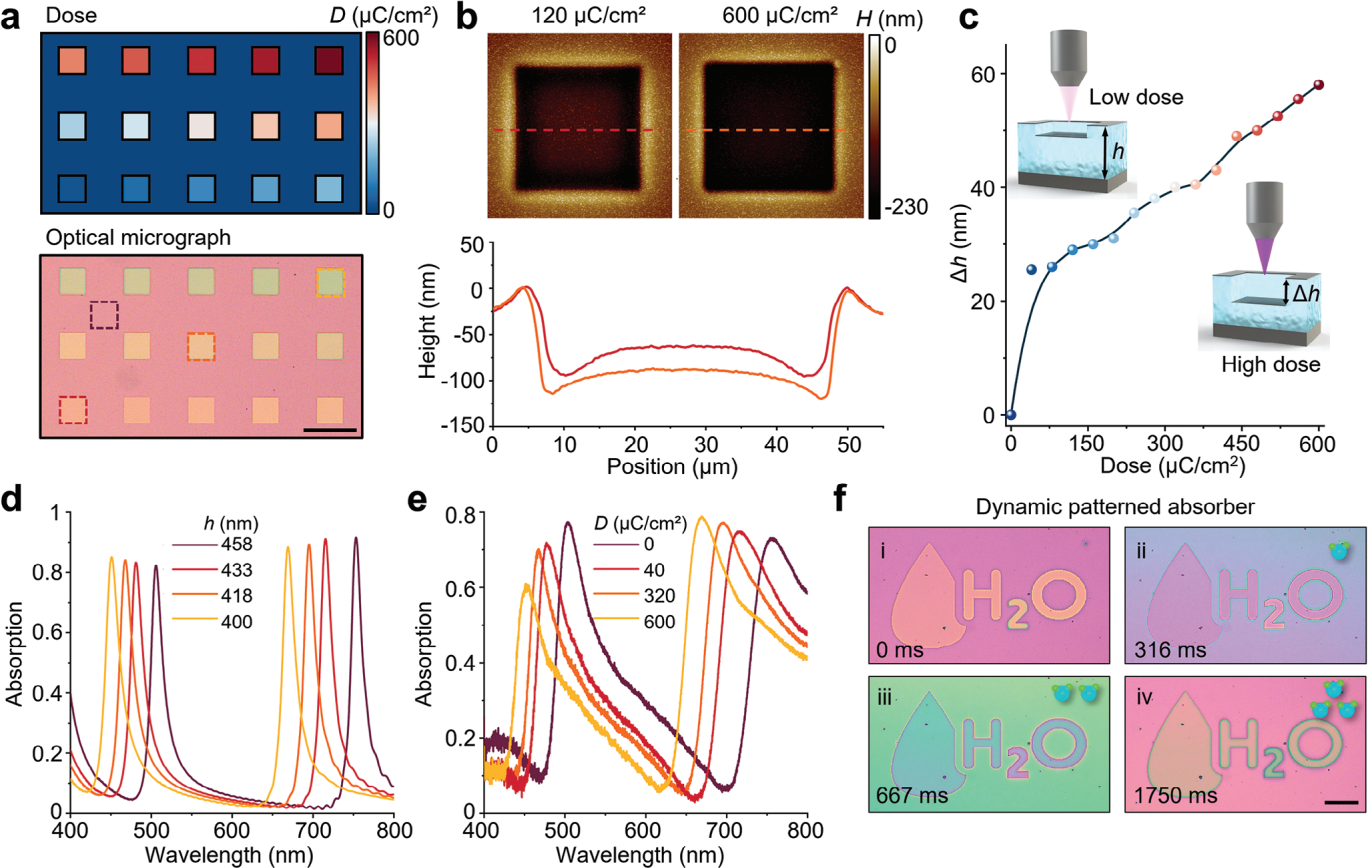
Fabrication of direct‐patterned hydrogel‐based absorber and its optical properties. a) Computer‐generated layout of patterns with exposure dose variation and the experimentally observed colors of the fabricated hydrogel absorber. Scale bar, 70 µm. b) Atomic force micrographs of the fabricated hydrogel nanocavity under the exposure dose of 120 and 600 µC cm^−2^, respectively. c) Measured hydrogel nanocavity shrinkage versus exposure dose at indoor humidity (RH ≈50%). d) Simulated absorption of hydrogel nanocavity with different hydrogel thicknesses. e) Corresponding measured absorption of fabricated hydrogel absorber with different exposure doses. f) Optical images of the direct‐printed hydrogel nanocavity with “H_2_O” pattern under multi‐level humidity tuning. Scale bar, 110 µm.

To experimentally prove the direct‐patterning technique on hydrogel absorbers, we fabricate hydrogel nanocavities as a series of individual square pixels (35 × 35 µm^2^) by using various exposure doses from 0 to 600 µC cm^−2^ (**Figure** **2**a). Because the absorption peak shifts from hydrogel shrinkage difference, the hydrogel nanocavity pixels exhibit different colors in the optical micrograph. The atomic force micrographs of the fabricated hydrogel nanocavity reconfirm the dose‐induced hydrogel shrinkage (Figure 2b). As the exposure dose increases to 600 µC cm^−2^, the height difference Δ*h* between the exposed hydrogel nanocavity and the unexposed nanocavity could reach ≈60 nm (Figure 2c). We further measure the absorption of fabricated hydrogel nanocavities, which is well‐aligned with the simulated results (Figure 2d,e). The increase in exposure dose leads to a decrease in the cavity length of the hydrogel absorber, resulting in a blue shift in the absorption peak. Note that the slight profile differences between measured and simulated spectra are caused by the roughness of the top Ag layer in actual fabrication. To experimentally demonstrate the feasibility of arbitrary direct‐patterning hydrogel absorbers, we fabricate the hydrogel nanocavity with an “H_2_O” pattern (Figure 2f). The “H_2_O” pattern is formed by micro‐pixels with a pixel size of 1.75 µm and a gap of 250 nm to avoid crosstalk from dose spread between adjacent pixels (see more details in Figures S1 and S2, Supporting Information). As the RH increases around the sample, the pattern color evidently and rapidly shifts within hundreds of milliseconds, indicating the dramatic tuning of absorption spectra.

**Figure 3:**
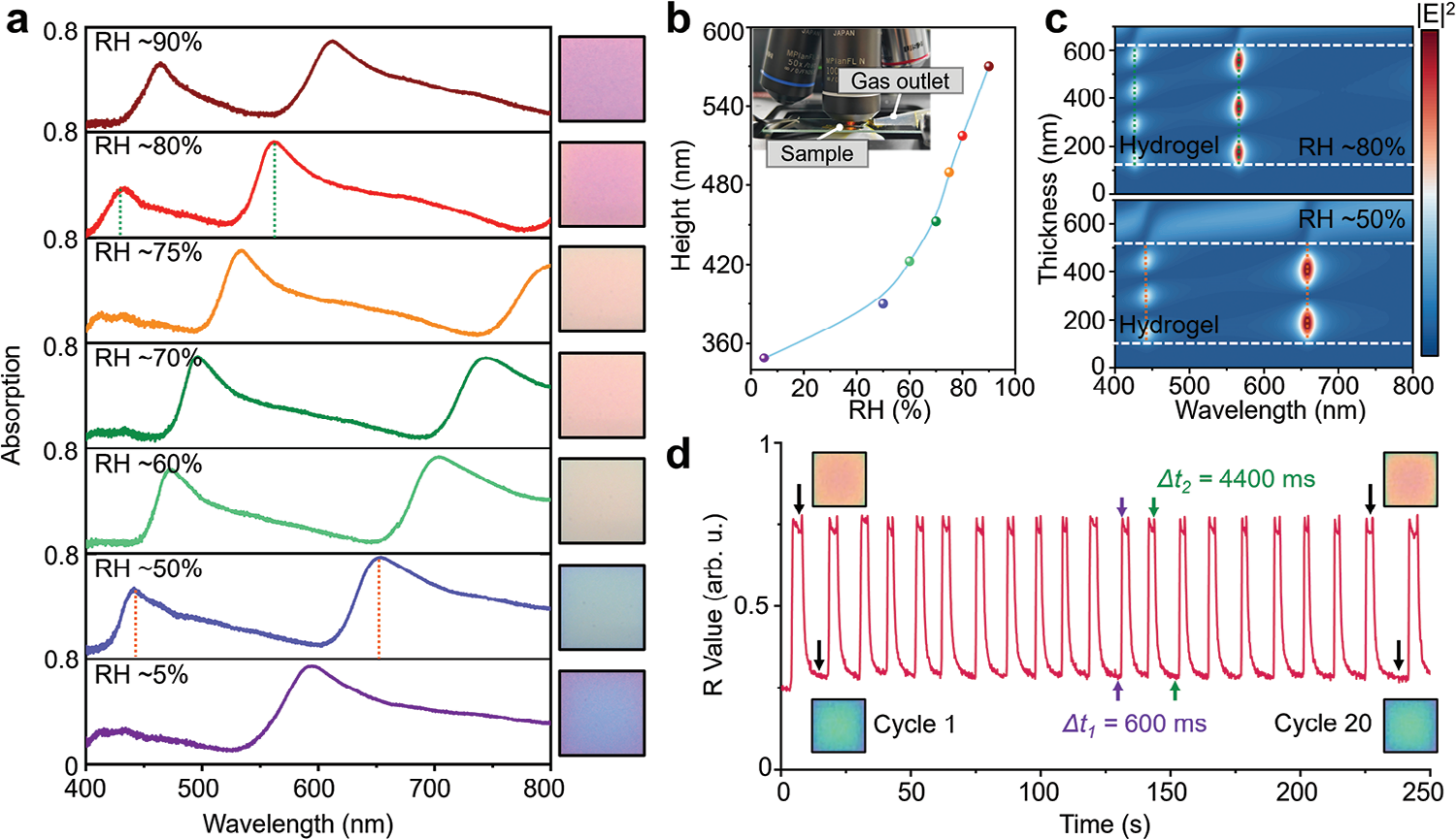
Optical measurement of tunable absorption. a) Measured absorption spectra and optical color of hydrogel absorber at RH ranged from ≈5% to ≈90%. b) The cavity height variation with the RH increase. The inset exhibits the experimental setup for humidity measurement. c) Simulated electric field distribution of hydrogel nanocavity with different hydrogel thicknesses, which corresponds to RH conditions of ≈50% and ≈80% in (a). d) The repeatability test of hydrogel nanocavity by recording the red component intensity during humidity adjustment between RH ≈50% and ≈75%.

To quantitatively study the humidity‐tunable optical performance of direct‐patterned hydrogel absorbers, we characterize the absorption spectra shift of a single hydrogel nanocavity pixel as the RH increases from ≈5% to ≈90% (**Figure** **3**a). The expansion of the hydrogel layer contributes to a tremendous red shift of the absorption peak wavelength, enabling continuously tunable absorption in visible frequency. Figure 3b shows the hydrogel layer height variation under different RH, revealing that the swelling ratio of hydrogel nanocavity could reach ≈1.6 folds when the RH increases from ≈5% to ≈90%. Since the spectral properties of the FP‐type resonator highly depend on the cavity length, the hydrogel layer height is numerically evaluated by retrieving the measured absorption spectra of hydrogel nanocavity. In the experiment, the ambient RH around samples is adjusted by blowing dry or wet nitrogen gas onto the sample using a gaseous injector, as shown in the inset.

To visualize the resonance alternation in hydrogel absorbers under different humidity conditions, we calculated the electric field intensity profile of nanocavity with the hydrogel thickness of 390 and 518 nm, which corresponds to the humidity conditions of RH ≈50% and ≈80%, respectively (Figure 3c). The E‐field is highly confined in the hydrogel layer at the resonance wavelengths, where a standing wave is formed between the top and bottom Ag layers due to constructive interference of incoming and reflected waves. To gain more insight into the repeatability and response speed of the hydrogel absorber, we conduct a cyclic humidity test of hydrogel nanocavity by repeatedly switching the RH around the sample between ≈50% and ≈75%, and record the red component variation of its optical color in real‐time (Figure 3d). After more than 20 cycles of humidity testing, the swelling behavior of the hydrogel absorber is not significantly affected, incidcating the robustness of hydrogel devices. By analyzing the red component value (R Value) change of the hydrogel nanocavity's color, the response time of the hydrogel absorber is ≈600 ms, and the recovery time is ≈4400 ms when applying/stopping the humidity stimulus (see more details in Figure S3, Supporting Information). Note that uneven intervals in the cycling test are caused by the manually triggered humidity regulation and can be improved by using a programmable control device.

**Figure 4:**
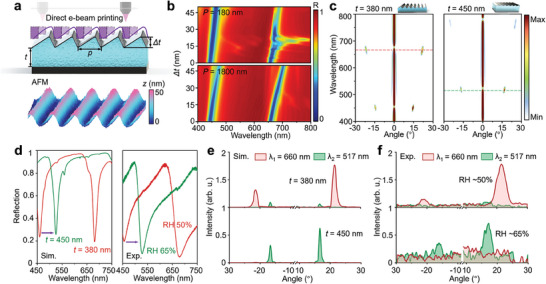
Dynamic wavelength‐selective beam steering. a) Schematic of the direct‐patterning fabrication of hydrogel metagrating using grayscale e‐beam lithography. The inset shows the atomic force micrographs of the fabricated 1800‐nm period hydrogel metagrating with a blazed‐grating profile. b) Simulated reflection spectra of hydrogel metagrating with thickness difference Δ*t* variation under the period of 180 and 1800 nm. c) Simulated the angle‐resolved diffraction of 1800‐nm period hydrogel metagrating under hydrogel thickness of *t* = 380 nm and 450 nm. The light illuminates the metagrating with normal incidence and arbitrary polarization. d) Measured reflection of hydrogel metagrating under the RH of ≈50% and ≈65%, corresponding to the simulated reflection with different hydrogel thicknesses. e) Line plots for corresponding diffraction intensity at the resonance wavelength in (c), as marked by dashed lines. f) Measured wavelength‐selective beam steering switching of metagrating under different humidity conditions.

To expand the light‐field manipulation capability of the hydrogel absorber, the hydrogel metagrating is fabricated by directly patterning the hydrogel nanocavity with a blazed grating profile (**Figure** **4**a). The atomic force micrograph (AFM) shows the morphology of fabricated metagrating with the 1800‐nm period (*p*), which exhibits a thickness difference Δ*t* of 25 nm due to the gradient dose distribution (see more details in Figure S4, Supporting Information). To investigate the effect of grating profile on the FP‐type cavity resonance, we simulate the reflection spectra of hydrogel metagrating as a function of Δ*t* under the periods of 180 and 1800 nm, respectively (Figure 4b). Compared to the hydrogel metagrating with a 180 nm period, the FP resonance of the 1800‐nm period metagrating is not strongly affected when Δ*t* varies from 0 to 50 nm. Since FP resonance is mainly determined by the cavity length, the reflection spectra of 1800‐nm period metagrating also deteriorate when Δ*t* is >75 nm (see more details in Figure S5, Supporting Information). To study the tunable optical performance of hydrogel metagrating, the angle‐resolved reflection is calculated with different hydrogel layer thicknesses *t* = 380 and 450 nm (Figure 4c). It can be observed that light near the resonant wavelength is deflected to the diffraction order, and other light is reflected vertically, which reveals the successful combination of FP resonance and grating effect. The diffraction angle *θ* of resonance wavelength can be calculated by the general law of grating reflection *θ* = arcsin(*mλ*/*p*), where *m* is the diffraction order, *λ* is the operating wavelength, and *p* is the period of grating. Moreover, the resonance shift from hydrogel layer expansion could effectively tune the operating wavelength of hydrogel metagrating, providing the feasible of dynamic wavelength‐selective beam steering.

To experimentally validate the dynamic properties of hydrogel metagrating, we characterize its tunable absorption and switchable beam steering capabilities under different humidity conditions. Figure 4d shows that the measured reflection of hydrogel metagrating is actively tuned by increasing ambient humidity from RH ≈50% to ≈65%, and the resonance peak shifts ≈70 nm, which is consistent with the simulated results, indicating that the hydrogel layer expands from 380 to 450 nm. Since the thickness difference of hydrogel metagrating is only ≈25 nm, the metagrating is observed with a uniform color under white illumination and its color dynamically shifts with humidity variation (see more details in Figure S7, Supporting Information). Based on the theoretical calculation in Figure 4c, when the hydrogel layer thickness swells from 380 to 450 nm, the operating wavelength of metagrating for beam steering is actively tuned from 660 to 517 nm, with the diffraction angle varying from 21.5° to 16.7° due to the grating dispersion, as plotted in Figure 4e. Figure 4f shows the experimentally measured dynamic wavelength‐selective diffraction of hydrogel metagrating as the RH increases from ≈50% to ≈65%, which is in excellent agreement with our design (see more details in Figure S8, Supporting Information). The diffraction intensity of the hydrogel metagrating switches between the wavelength of 660 and 517 nm with high contrast due to the FP resonance. Since the hydrogel layer swelling is continuous, the metagrating could achieve arbitrary wavelength‐selective diffraction between the operating wavelength channels by more precise control of the ambient humidity. The experiment adopts unpolarized light to illuminate the sample at normal incidence because the mategraing exhibits polarization‐independent properties (see more details in Figure S9, Supporting Information). In addition, when the RH increases to ≈75%, the hydrogel layer thickness swells to 500 nm, and the resonant wavelength of metagrating could be further tuned to 564 nm. However, the excessive hydrogel swelling would lead to a reduction in thickness difference of metagrating, resulting in a decrease in diffraction intensity (see more details in Figures S10 and S11, Supporting Information). In other words, the morphology alternation of hydrogel metagrating may serve as an efficiency method to continuously manipulate grating efficiency.

**Figure 5:**
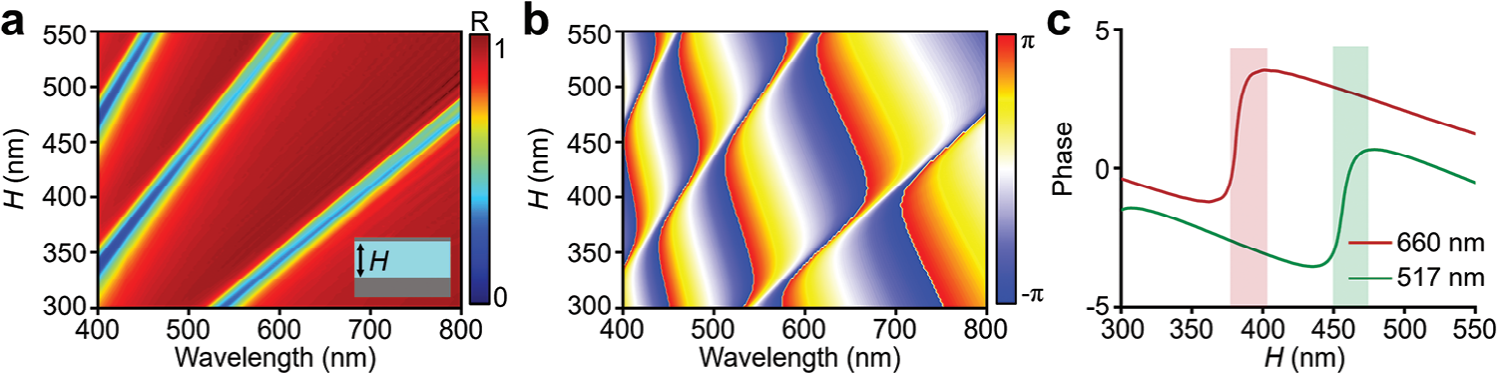
Phase modulation ability of FP‐type hydrogel nanocavity. a) Simulated reflection of hydrogel absorber as core layer height *H* increases. b) Phase delay of hydrogel nanocavity as a function of wavelength and *H*. c) Corresponding line plot of phase delay with varied *H* at the wavelength of 517 and 660 nm.

To gain more insight into the basic principle of hydrogel metagrating, we study the phase modulation ability of FP‐type nanocavity, as shown in **Figure** **5**. As the hydrogel layer height *H* increases from 300 to 550 nm, the resonance peak is continuously tuned, and higher resonant modes emerge due to the cavity effect (Figure 5a).^[^ ^42^ ^]^ Figure 5b shows that hydrogel nanocavity exhibits drastic phase alteration around the resonance peak, indicating the phase modulation ability of height‐varied FP structures. To compare the phase modulation difference of metagrating under different RH conditions, Figure 5c plots the phase delay as a function of *H* at 660 and 517 nm wavelengths, corresponding to the wavelength of beam steering switching in Figure 4. When the hydrogel nanocavity height *H* ranges from 380 to 405 nm, the phase delay shows an intensive shift at 660 nm wavelength and a steady change at 517 nm, revealing the working mechanism of wavelength‐selective diffraction from metagrating. While *H* ranges from 450 to 475 nm, the tendency of phase shift is completely opposite, which is in good agreement with the switchable optical performance of metagrating. The FP‐type nanocavity provides considerable phase modulation within a height variation of <30 nm, enabling the wavefront shaping ability of hydrogel metagrating.

## 3 Conclusion

In summary, we experimentally demonstrate hydrogel‐based nanocavity and metagrating using direct‐patterning technology for humidity‐tunable absorption and wavelength‐selective beam steering. Through exploiting the polymer shrinkage under high‐energy irradiation, the hydrogel nanocavity could be directly printed with an arbitrary pattern. Furthermore, by applying the varied humidity onto the sample, the absorption peak of hydrogel nanocavity is continuously tuned with a rapid response speed (< 320 ms) and a wide shift range (> 150 nm) due to the hydrogel volume swelling. Moreover, by patterning the hydrogel nanocavity with a blazed grating profile, we demonstrate a hydrogel metagrating that combines the FP resonance and grating effect. Under white light illumination, the metagrating only deflects light at the resonant wavelength to the diffraction order, and the resonant wavelength could be actively tuned by regulating RH, enabling dynamic wavelength‐selective beam steering. Our proposed strategy for active absorption and diffraction tuning based on hydrogel materials could serve as a promising guideline of tunable metasurfaces for potential applications, including optical display, gas sensing, and spectral analysis.

## 4 Experimental Section

### Numerical Simulations

The finite‐difference time‐domain (FDTD) method was employed to calculate the absorption of hydrogel nanocavity and the angle‐resolved far‐field distribution of the hydrogel metagrating. The plane wave source at normal incidence with arbitrary polarization. Since the hydrogel nanocavity and metagrating are both periodic structures, the periodic boundary conditions were used in the *x*‐*y* plane in the 3D simulations. The perfectly matched layers were set along the propagation direction (*z*‐axis). The complex refractive index of Ag was utilized from the data of Palik (0–2 µm). The refractive index of PVA hydrogel was set as a constant 1.51 in the simulation since the refractive index variation during the hydrogel swelling is <0.1.^[^ ^13^ ^]^

### Sample Fabrications

The Ag‐PVA‐Ag three‐layer films were successively deposited on a silicon substrate to form the hydrogel nanocavity. The top Ag layer was deposited by thermal evaporation with an evaporation rate of 0.2–0.3 Å s^−1^. This evaporation rate was employed to ensure that the Ag film is fabricated with a certain roughness, which can facilitate the exchange of water molecules between the hydrogel layer and the surrounding environment. For the hydrogel layer, the diluted PVA solution was spin‐coated onto the bottom Ag layer at the speed of 4000 rpm for 60 s to give a ≈400 nm thick. Regarding the direct patterning of hydrogel absorber and metagrating, the samples were exposed by an e‐beam lithography system (Raith eLINE Plus, electron acceleration voltage of 10 kV).

## Conflict of Interest

The authors declare no conflict of interest.

## Supporting information



Supporting Information

## Data Availability

The data that support the findings of this study are available from the corresponding author upon reasonable request.
