# Supplementary material for: Dynamic Wavelength‐Selective Diffraction and Absorption with Direct‐Patterned Hydrogel Metagrating
Source: Adv Sci (Weinh). 2024 Oct 17;11(46):2408960. doi: 10.1002/advs.202408960 (PMC11633533; doi:10.1002/advs.202408960)
Supplement: Supplementary file 1 — Supporting Information [file ADVS-11-2408960-s001.pdf]

ADVANCED SCIENCE
Open Access

Supporting Information

for *Adv. Sci.*, DOI 10.1002/advs.202408960

Dynamic Wavelength-Selective Diffraction and Absorption with Direct-Patterned Hydrogel Metagrating

Chenjie Dai, Xinglong Li, Wen-xing Yang*, Yan Chen, Dingshan Zheng, Nian Cheng, Tao Shui, Huafeng Zhang and Zhongyang Li**

Supporting Information

Dynamic wavelength-selective diffraction and absorption with direct-patterned hydrogel metagrating

Chenjie Dai, Xinglong Li, Wen-xing Yang*, Yan Chen, Dingshan Zheng, Nian Cheng, Tao Shui, Huafeng Zhang, and Zhongyang Li**

1. Measured reflection of direct-patterned hydrogel absorber

In the experiment, the "H_2O" pattern is formed by micro-pixels. The pixel size is 1.75 μm with a 250-nm gap to avoid crosstalk from dose spread between adjacent pixels, as shown in Figure S1. The captured optical image of the "H_2O" pattern shows a uniform color of each part and is not strongly affected by spacing due to the low duty ratio. In this design, the varied exposure doses are employed to pattern the different areas of "H_2O" pattern, as shown in Figure S2. Therefore, there is a difference in the color of each part, which would further increase as the hydrogel expands.[1]

For the slight color difference of "H_2O" pattern background under different humidity conditions (Figure 2f), this is because the humid gas is applied to the sample on one side, as shown in Figure 3b. The slightly uneven humidity distribution on the sample surface causes the expansion difference of hydrogel nanocavity, which results in the nonuniformity of the color. The expansion difference from uneven humidity distribution occurs on a large scale and would not affect the dynamic optical measurement of the hydrogel absorber and metagrating. We envision that this humidity test could be improved by encapsulating our sample in to a microfluidic system for

stable humidity control.

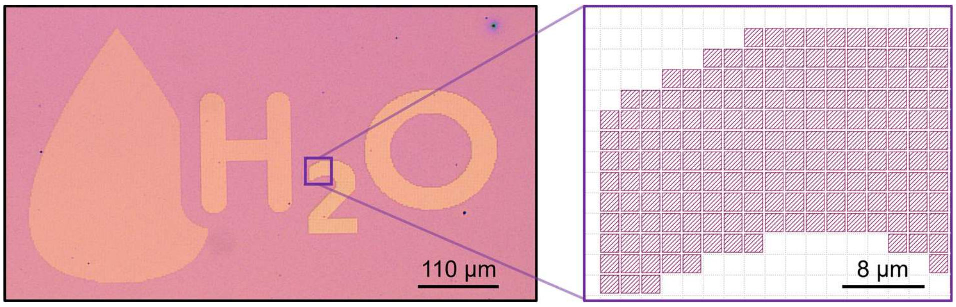

Figure S1. Schematic of the pixel distribution of "H_2O" pattern.

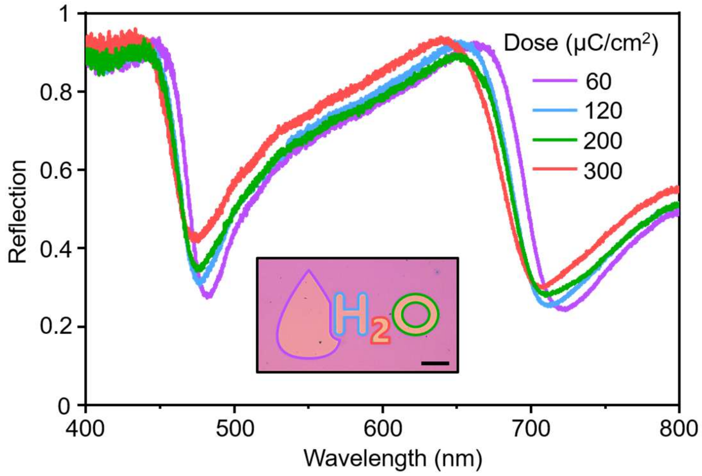

Figure S2. Measured reflection of hydrogel nanocavities at different areas of the "H_2O" pattern. Scale bar, 110 μm.

2. Repeatability test of hydrogel nanocavity

The zoom-in plot of repeatability test is plotted to further show the humidity response properties of the hydrogel absorber in detail (Figure S3). The hydrogel nanocavity rapidly responds when a high humidity stimulus is applied to the sample and exhibits a steady recovery process when the humidity treatment is stopped. The response time could be further improved by optimizing the top Ag layer thickness and evaporation

process.[2]

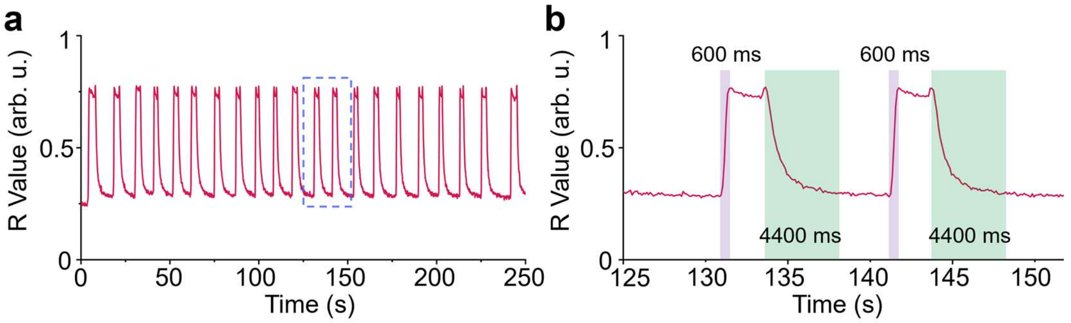

Figure S3. (a) The repeatability test of hydrogel nanocavity by recording the red component intensity during humidity adjustment. (b) The zoom-in plot of humidity response in (a).

3. Atomic force micrographs of the hydrogel metagrating

The 3D AFM image of fabricated metagrating in Figure 4a is measured by high-resolution AFM (Dimension icon, Germany Bruker). To further visualize the morphology of metagrating, we demonstrate the 2D AFM image and the line plot of morphology variation, as shown in Figure S4. The metagrating exhibits a thickness difference $\Delta t \sim 25$ nm, which could provide sufficient phase modulation for wavelength-selective beam steering due to the drastic phase shift from resonance (Figure 5). At the current stage, the hydrogel grating could be fabricated with good morphology at a minimum period of 1200 nm using the electron-beam direct-patterning technique,[3] since the PVA hydrogel is not a typical photoresist. We envision that the hydrogel metagrating could be fabricated with a smaller grating period by optimizing the processing or adopting other fabrication technologies, such as nanoimprinting.[4]

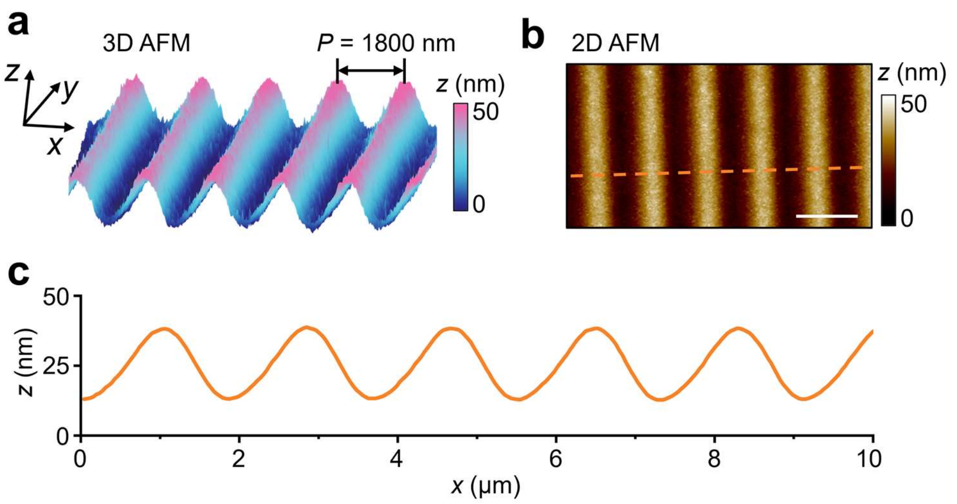

Figure S4. (a, b) 3D and 2D atomic force micrographs of fabricated 1800-nm period metagrating. Scale bar, 2 μm. (c) Line plot of the morphology variation of metagrating.

4. The effect of thickness difference on the resonance of metagrating

To visualize the effect of thickness difference Δt on the resonance of metagrating, the electric field profile is simulated under different Δt at corresponding resonant wavelengths, as shown in Figure S5. Based on the working principle of FP-type nanocavity, the electric field is highly confined at the hydrogel layer, and a standing wave is formed at the resonant wavelength due to the interference. When Δt is 25 nm, the reflection spectra are not strongly affected, and the electric profile of the standing wave is relatively uniform. When Δt is larger than 75 nm, the electric field enhancement occurs in a part of metagrating since FP resonance is mainly determined by the cavity length. The increase of cavity length difference Δt allows the metagrating to exist in more resonance modes, which makes the reflection spectra more complicated.

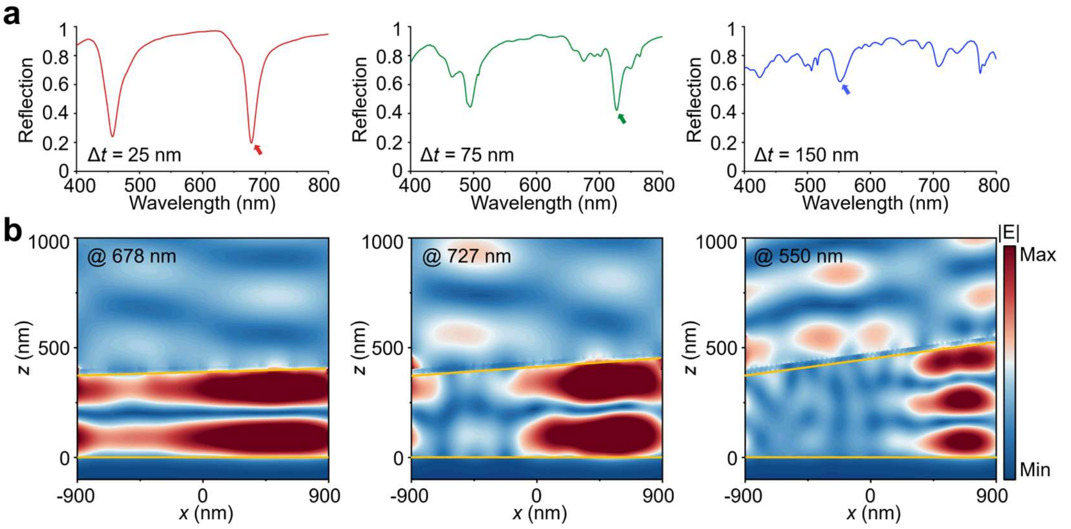

Figure S5. (a) Simulated reflection spectra of metagrating with different hydrogel layer thickness difference Δ*t*. The grating period is 1800 nm, and the hydrogel layer thickness is 380 nm. (d) Corresponding electric field profile of metagrating with a unit cell area at resonant wavelengths in (a).

5. Eletric field profile of metagrating for beam steering

The phase delay of metagrating is accumulated along the *x*-direction in the period as the hydrogel thickness increases (Figure 5), thus deflecting the beam to a positive angle.[5] To visualize the direction of diffraction light, we simulate the electric field profile of metagrating (Figure S6), confirming that the metagrating steers light of normal incidence to a positive angle.

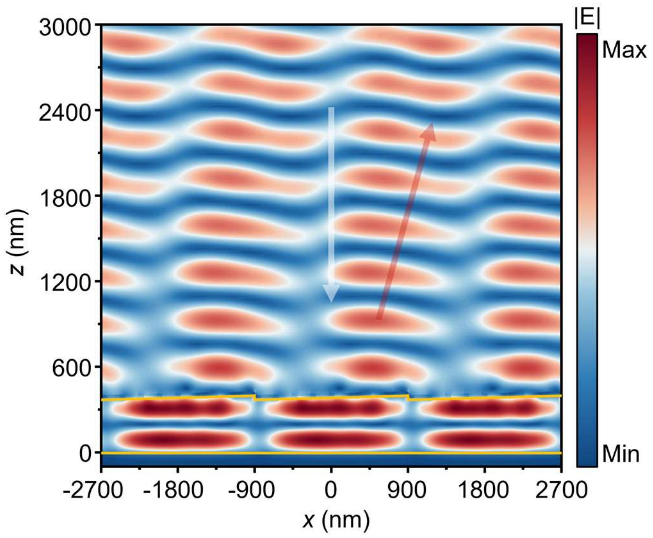

Figure S6. Simulated electric field profile of metagrating for beam steering at operating wavelength of 660 nm.

6. Photograph of the dynamic color shift of the hydrogel metagrating

As shown in Figure S7, it is experientially observed that the reflective color of the fabricated hydrogel metagrating dynamically and uniformly shifts.

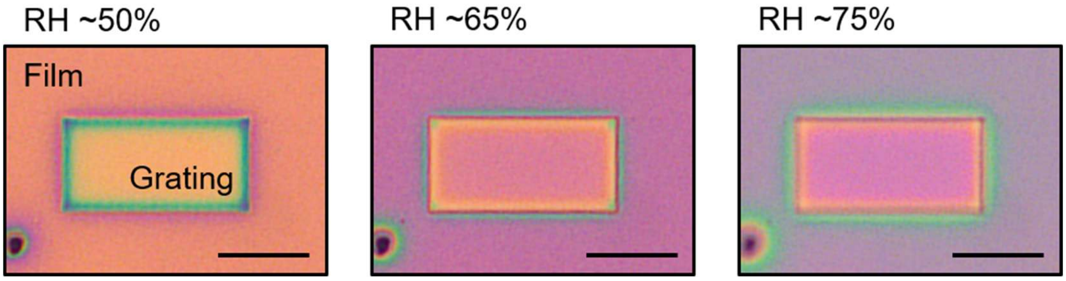

Figure S7. Observed the dynamic reflective color shift of the hdyrogel metagrating as the RH increases from ~ 50% to ~ 75%. Scale bar, 50 μm.

7. Diffraction efficiency of metagrating as a function of wavelength

To visualize the wavelength-selective properties of the diffraction order, we calculate the diffraction efficiency of the +1 order under different humidity conditions, which is well-aligned with the simulation, as shown in Figure S8. Because our measurement system has a weaker response at short wavelengths, the diffraction efficiency at operating wavelengths of 450 nm and 660 nm exists a slight difference between simulation and experiment. Both experimental and simulation results reconfirm that the metagrating exhibits wavelength-selective properties for beam steering.

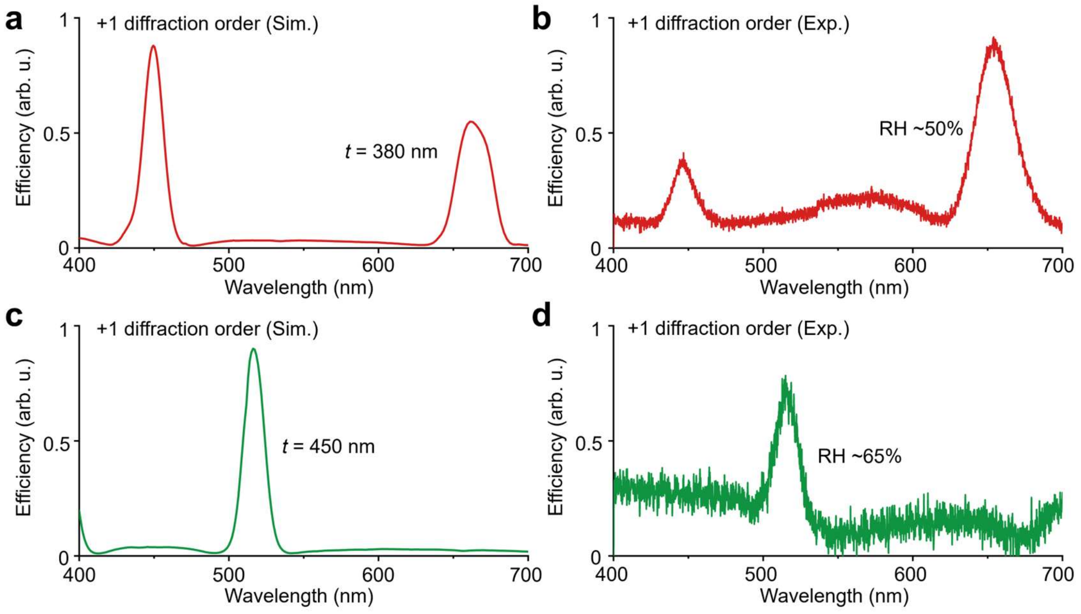

Figure S8. Simulated and experimentally calculated efficiency of +1 diffraction order at different hydrogel thickness, corresponding to the RH of (a, b) ~50% and (c, d) ~65%.

8. Polarization dependence properties of metagrating

Regarding the polarization dependence of metagrating, we measured the angle-resolved diffraction of metagrating under *x*- and *y*-polarized illumination using a polarizer, which is in good agreement with the simulation results, as shown in Figure S9. Both simulation and experimental results show the polarization-independent characteristic of metagrating. Therefore, we mainly adopt the unpolarized light to illuminate the sample in the experiment.

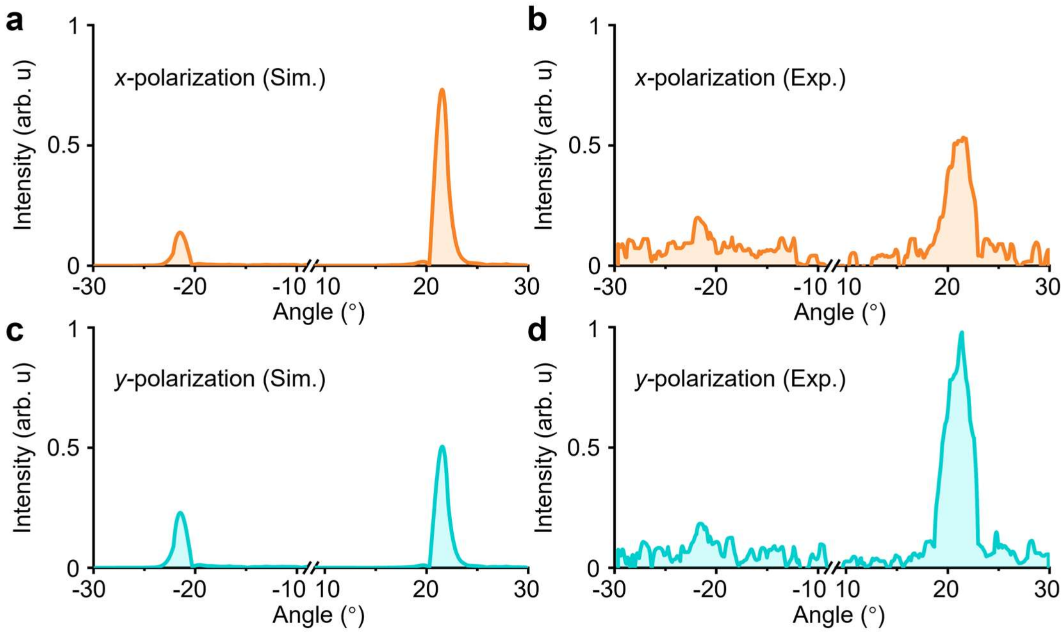

Figure S9. Simulated and measured diffraction intensity for (a, b) *x*-polarization and (c, d) *y*-polarization at the operating wavelength of 660 nm, corresponding to the hydrogel thickness of 380 nm and the RH of ~50%.

9. Humidity-responsive tunable optical properties of the hydrogel metagrating

For low relative humidity conditions (RH ~5%), the resonance wavelength of metagrating exhibits a slight blue shift due to the hydrogel shrinkage (Figure S10a). Since the hydrogel exhibits a low expansion scaling when relative humidity is below 50% (Figure 3b), the operating diffraction wavelength alters from 660 nm to 640 nm as the RH decreases from 50% to 5%, corresponding to the variation in hydrogel layer thickness from 380 nm to 370 nm (Figures 4 and S10b). For higher humidity conditions, we measured the reflection of metagrating under the RH of ~70%, which corresponds to the hydrogel layer thickness of 488 nm. Moreover, as the RH increases to ~75%, the resonant peak wavelength of metagrating shifts to 564 nm (Figure S10c), corresponding to the hydrogel layer thickness of 500 nm retrieved from the simulation. Due to the grating efficiency decrease from hydrogel metagrating morphology alternation (see more details in Figure S11), the hydrogel metagrating deflects the light at the resonant wavelength of 564 nm to the diffraction order with weak intensity contrast. The excessive inflation of the hydrogel layer would strongly weaken the thickness difference Δt of metagrating, resulting in the grating efficiency vanishing[3]. Therefore, we demonstrate the dynamic wavelength-selective beam steering under the RH of 50% and 65% to ensure optical performance.

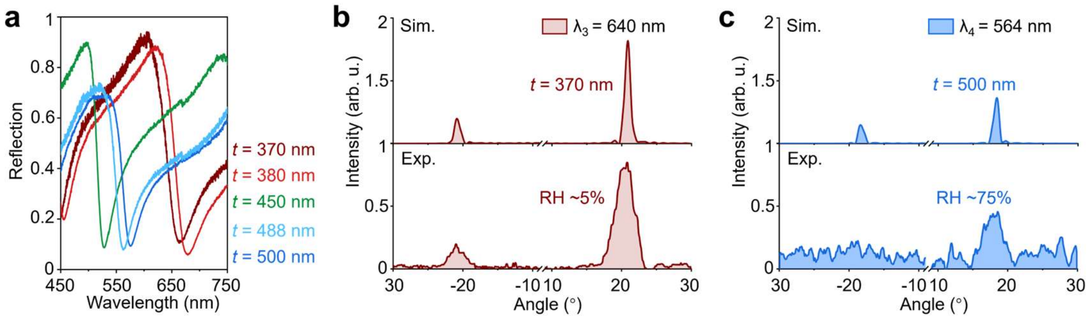

Figure S10. (a) Measured reflection of hydrogel metagrating under different humidity conditions. (b, c) Simulated and measured diffraction intensity of hydrogel metagrating at resonant wavelengths under the RH of ~5% and ~75%.

10. Diffraction intensity versus thickness difference of hydrogel grating

Due to the phase modulation from cavity resonance, the metagrating exhibits favorable beam steering capability when the thickness difference Δ*t* between 25 nm and 35 nm (Figure S11a). Figure S11b shows that the diffraction intensity of hydrogel grating reduces to zero when the thickness difference Δ*t* decreases from 25 nm to 0 nm. Therefore, in the humidity test of metagrating, the hydrogel swelling induces the Δ*t* to decrease, leading to the diffraction efficiency reduction under the high RH (>75%).

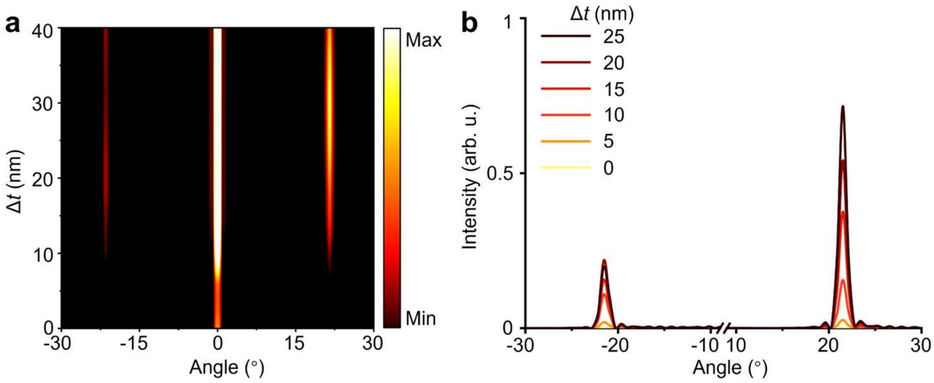

Figure S11. (a) Simulated diffraction intensity of hydrogel metagrating with varied thickness different Δ*t* at the resonant wavelength of 660 nm. (b) Corresponding line

plots of diffraction intensity variation with the Δt decreases from 25 nm to 0 nm. The hydrogel layer thickness is set at 380 nm.

11. Angular selectivity of metagrating

For the angular selectivity of metagrating, we simulated the reflection and angle-resolved diffraction spectra of metagrating under different incident angles, as shown in Figure S12. Due to the cavity-induced wavelength sensitivity,[6] the resonance peak wavelength blue shifts as the incident angle increases. Since the height difference Δt is small, the resonance difference is not obvious at positive and negative incident angles. As for the far-field diffraction, the diffraction order remains the wavelength selectivity properties under different incident angles. The diffraction angle shifts with the incident angle change, and the diffraction intensity gradually decreases under a large incident angle due to weakened resonance from the deterioration of interference conditions.

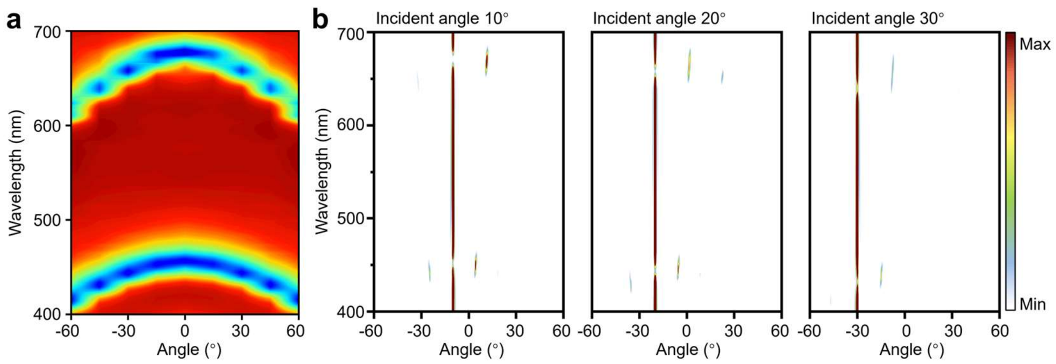

Figure S12. (a) Simulated reflection of 1800-nm period metagrating under hydrogel thickness of t = 380 nm at different incident angles. (b) Simulated angle-resolved diffraction at corresponding incident angles.

References

[1] C. Dai, Z. Li, Z. Li, Y. Shi, Z. Wang, S. Wan, J. Tang, Y. Zeng, Z. Li, *Adv. Funct. Mater.* **2023**, 33, 2212053.

[2] C. Jung, S.-J. Kim, J. Jang, J. H. Ko, D. Kim, B. Ko, Y. M. Song, S.-H. Hong, J. Rho, *Sci. Adv.* **2022**, 8, eabm8598.

[3] C. Dai, S. Wan, Z. Li, Y. Shi, S. Zhang, Z. Li, *Nat. Commun.* **2024**, 15, 845.

[4] B. Ko, N. Jeon, J. Kim, H. Kang, J. Seong, S. Yun, T. Badloe, J. Rho, *Microsyst. Nanoeng.* **2024**, 10, 1.

[5] Z. Li, E. Palacios, S. Butun, K. Aydin, *Nano Lett.* **2015**, 15, 1615.

[6] C. Dai, Z. Li, Y. Shi, S. Wan, W. Hu, Z. Li, *Laser Photonics Rev.* **2023**, 17, 2200368.
